# COX-2, CB2 and P2X7-immunoreactivities are increased in activated microglial cells/macrophages of multiple sclerosis and amyotrophic lateral sclerosis spinal cord

**DOI:** 10.1186/1471-2377-6-12

**Published:** 2006-03-02

**Authors:** Yiangos Yiangou, Paul Facer, Pascal Durrenberger, Iain P Chessell, Alan Naylor, Chas Bountra, Richard R Banati, Praveen Anand

**Affiliations:** 1 Peripheral Neuropathy Unit, Imperial College, Hammersmith Hospital, London, UK; 2 GastrointestinaI Diseases Centre of Excellence for Drug Discovery, GlaxoSmithKline, Harlow, UK; 3 School of Medical Radiation Sciences and Ramaciotti Centre for Brain Imaging, Brain-Mind Research Institute, University of Sydney, New South Wales, Australia

## Abstract

**Background:**

While multiple sclerosis (MS) and amyotrophic lateral sclerosis (ALS) are primarily inflammatory and degenerative disorders respectively, there is increasing evidence for shared cellular mechanisms that may affect disease progression, particularly glial responses. Cyclooxygenase 2 (COX-2) inhibition prolongs survival and cannabinoids ameliorate progression of clinical disease in animal models of ALS and MS respectively, but the mechanism is uncertain. Therefore, three key molecules known to be expressed in activated microglial cells/macrophages, COX-2, CB2 and P2X7, which plays a role in inflammatory cascades, were studied in MS and ALS post-mortem human spinal cord.

**Methods:**

Frozen human post mortem spinal cord specimens, controls (n = 12), ALS (n = 9) and MS (n = 19), were available for study by immunocytochemistry and Western blotting, using specific antibodies to COX-2, CB2 and P2X7, and markers of microglial cells/macrophages (CD 68, ferritin). In addition, autoradiography for peripheral benzodiazepine binding sites was performed on some spinal cord sections using [3H] (R)-PK11195, a marker of activated microglial cells/macrophages. Results of immunostaining and Western blotting were quantified by computerized image and optical density analysis respectively.

**Results:**

In control spinal cord, few small microglial cells/macrophages-like COX-2-immunoreactive cells, mostly bipolar with short processes, were scattered throughout the tissue, whilst MS and ALS specimens had significantly greater density of such cells with longer processes in affected regions, by image analysis. Inflammatory cell marker CD68-immunoreactivity, [3H] (R)-PK11195 autoradiography, and double-staining against ferritin confirmed increased production of COX-2 by activated microglial cells/macrophages. An expected 70-kDa band was seen by Western blotting which was significantly increased in MS spinal cord. There was good correlation between the COX-2 immunostaining and optical density of the COX-2 70-kDa band in the MS group (r = 0.89, P = 0.0011, n = 10). MS and ALS specimens also had significantly greater density of P2X7 and CB2-immunoreactive microglial cells/macrophages in affected regions.

**Conclusion:**

It is hypothesized that the known increase of lesion-associated extracellular ATP contributes via P2X7 activation to release IL-1 beta which in turn induces COX-2 and downstream pathogenic mediators. Selective CNS-penetrant COX-2 and P2X7 inhibitors and CB2 specific agonists deserve evaluation in the progression of MS and ALS.

## Background

Multiple sclerosis (MS) is a chronic, immune-mediated disorder of the central nervous system. MS patients may be affected by a relapsing-remitting form of the disease, but a large proportion of patients will progress to a secondary progressive form of the disease, which results in a gradual and progressive loss of neurological function. Progression of neurological dysfunction is also a characteristic of amyotrophic lateral sclerosis (ALS), a neuro-degenerative motor disorder with poor prognosis. While new treatments have shown some efficacy in ALS and MS [1,2], more effective therapies are required to slow progression and reduce disability and mortality. As there is increasing evidence for shared cellular mechanisms that may affect disease progression in CNS disorders, particularly glial responses, we have studied the expression of key mechanisms in the neuro-inflammatory cascade, COX-2, CB2 and P2X_7_, in MS and ALS post-mortem human spinal cord.

There are two major forms of cyclooxygenase (COX), the iso-enzymes, COX-1 and COX-2 [3]. COX-1 is constitutively synthesized in a variety of tissues including gastric mucosa, liver, kidneys, and platelets where, prostaglandin production in these sites maintains normal tissue function. COX-2 is an inducible enzyme that is present in low amounts in normal adult tissues but is increased in peripheral and central nervous system and in monocytes following injury or inflammation [4]. Gene targeting techniques have been used to create strains of "knockout" mice that lack COX-2. These strains have frequent reproductive failures, kidney dysfunction, and a shortened life span [5]. Reproductive failure appears related to specific COX-2-, but not COX-1-derived prostaglandin that is essential for placental formation and maintenance [5]. Carrageenan induces inflammation in COX-2-deficient mice, and these inflammatory responses can be suppressed in part by COX-1 inhibition, suggesting that COX-1 may also mediate inflammation in these animals.

COX-2 expression is increased by a number of pro-inflammatory cytokines, including interleukin 1 and tumor necrosis factor alpha, as well as by other factors, including endotoxin, hypoxia, ischemia, epidermal growth factor and transforming growth factor beta 1. COX-2 expression is increased in spinal cord neurons following peripheral inflammation [6]. Inflammation produces robust increases in COX-2 expression diffusely in the rat brain, especially in and around blood vessels. Prostaglandins derived from COX-2 expression in cerebral vessels appear important in the generation of fever. Focal or global cerebral ischemia dramatically induces COX-2 expression [7]. Inhibition of both COX-1 and COX-2 may contribute to spinal analgesic and anti-hyperalgesic actions of non-steroidal anti-inflammatory drugs [8]. COX-2 inhibitors have also recently been suggested [9] as possible alternatives to glucocorticoids in the treatment of peritumoral edema in patients with malignant brain tumours, as they showed that glioma-infiltrating microglia are a major source of PGE_2 _production through the COX-2 pathway.

Recently, COX-2 mRNA was shown to be up-regulated in ALS spinal cord [10,11]. COX-2 inhibitors have been shown to have a therapeutic role in a transgenic mouse model of ALS [12]. These authors showed that prophylactic administration of the preferential COX-2 inhibitor, nimesulide, in the feed resulted in a significant delay in the onset of ALS type motor impairment.

Marijuana has been used for at least 4000 years as both a therapeutic agent and a recreational drug and the psychoactive ingredient is Δ^9^-tetrahydrocannabinol (THC). It is known that THC binds to receptors in the CNS known as CB1 and that a second subtype of these receptors CB2 are mainly present in the periphery in the normal state, particularly on macrophage cells of the immune system [13].

Actions of cannabinoids at peripheral cannabinoid receptors may explain altered immune function after long-term cannabinoid administration. Cannabinoids acting at CB2 receptors in the immune system cause inhibition of T-cell-dependent humoral immune responses through direct inhibition of accessory T-cell function [14]. Anandamide (arachidonoylethanolamide, AEA) and 2-arachidonoylglycerol (2-AG) are endogenous ligands of cannabinoid receptors [15]. The endocannabinoid system (the cannabinoid receptors and their endogenous ligands) seems to play a role in neuroprotection, as suggested by the up-regulation of CB_1 _receptor expression and the elevated concentrations of endocannabinoids in some in vivo models of neurodegeneration and brain injury [16]. Recent findings have shown that cannabinoids can down-regulate the production of nitric oxide (NO), in macrophages [17] and also in rat microglial and glial cells. Activated glial cells have also been proposed to play an active role in many neurodegenerative pathologies including MS and ALS [18,19] through the production and release of several pro-inflammatory and cytotoxic mediators including nitric oxide.

P2X_7 _is the most recently cloned member of the ATP-activated ligand-gated ion channel superfamily [20], and has been shown to cause externalisation of mature, biologically active IL-1β [21]. In addition, ATP is able to act in an autocrine manner when released from cells challenged by inflammatory initiators (e.g. LPS, [22], where P2X_7 _antagonists impair release of IL1β in mice lacking the P2X_7 _gene, release of mature IL-1β in response to LPS and ATP is absent [23]. Release of IL-1β leads to up-regulation of gene products which contribute to the inflammatory state, including matrix metalloproteinase's, COX-2, interleukins, and cellular adhesion molecules [24]. This pivotal role of the P2X_7 _receptor in the inflammatory cascade is hypothesised to be triggered by release of ATP from cells undergoing apoptosis or necrosis in neurodegenerative and neuroinflammatory processes, as it is well established that even mild perturbation of cells can cause ATP release [25]. The predominant expression of P2X_7 _is thought to be in immune cells including macrophages and microglial cells/macrophages [26], candidate cell populations for contribution to neurodegeneration observed in MS and ALS. Thus, it may be hypothesized that persistent activation of P2X_7 _may play a role in the pathogenesis of these neurodegenerative conditions.

In this study we show, by using immunochemical methods, that COX-2, CB2 and P2X_7 _immunoreactivity are localised mainly in activated microglial cells/macrophages in ALS and also MS cord, and that it is co-localised with the microglial cell/macrophage markers CD68, [26], and ferritin [27]. In addition, 3 [H] PK11195 autoradiographic binding studies [28] has been used to further corroborate regions of microglial cell/macrophage activation, in comparison with the above markers.

## Methods

### Spinal cord

Segments of deep frozen human spinal cord were obtained from the rapid autopsy system of the Netherlands Brain Bank (NBB) and MRC Brain Bank, King's College London, from a total pool of controls (n = 12), ALS (n = 9) and MS (n = 19), for study by immunocytochemistry, Western blotting and autoradiography for peripheral benzodiazepine binding sites. All MS tissues were classified according to the following criteria: 1. preactive lesions – expression of CD68, CD 45 on clusters of perivascular microglial cells in white matter with no loss of myelin, 2. active demyelinating lesions, characterized by the presence of macrophages with luxol fast blue or myelin basic protein (MBP) – positive inclusions, and GFAP-positive reactive astrocytes with long processes, in demyelinating regions 3. Chronic active – hypocellular demyelinated centre of the lesions containing a small number of CD 68-postive macrophages, with reactive astrocytes localized mainly at the edge of the lesion, or 4. Chronic non-active lesions – hypocellular demyelinated lesion with gliosis (as described previously, validated by MRI-guided sampling) [29,30]. ALS lumbar spinal cord was obtained post-mortem from patients with clinically and pathologically confirmed sporadic ALS (Mean disease duration was 34 months, ranging from 7–84 months; mean age 67 years, range 51–76 years). The post-mortem delay for ALS patients ranged from 4 to 24 h, and for control subjects from 14 to 26 h.

### Immunocytochemistry

Frozen, transverse sections (12 μm) of spinal cord were collected onto poly- L – lysine coated slides and post-fixed in freshly prepared, 4% w/v paraformaldehyde in PBS for 30 min. Sections were then dehydrated through graded alcohols and endogenous peroxidase blocked by incubation with 0.3% w/v hydrogen peroxide for 30 min. After re-hydration and washing in PBS, sections were incubated overnight with primary antibody (Table 1). Sites of primary antibody attachment were revealed using avidin-biotin peroxidase method (Vector Labs UK – ABC – black product) and nuclei were counterstained in 0.1% w/v Neutral Red to give contrasting pink/red nuclei but not in double labelling experiments to avoid confusing colours. For CB2 specificity studies, experiments were performed with the primary antibody (Santa Cruz S-16 only, at a titre of 4 K) incubated with antigenic peptide (20, 2 and 0.2 μg/ml) for 2 hours prior to incubation with the sections. Peptide was not available for specificity studies for COX-2 and P2X_7_, but other methodological controls were performed (omission of primary antibodies, sequential dilution of antibodies).

**Table 1:** Antibodies used in this study

Antibody	**Donor**	Source	**Ref**	**Dilution**
Mouse COX-2	Mouse	BD Biosciences, Oxford UK.	C22420	1/750
Mouse P2X_7_	Mouse	Geneva Biomedical Research Institute	EL101116/122 L4	1/4000
Ferritin	Rabbit	Abcam, Cambs. UK	Ab7332	1/1000
CD2	Mouse	Dako, High Wycombe, UK	M0720	1/100
CD68	Mouse	Dako, High Wycombe, UK	EBM11	1:500
CB2	Rabbit	GSK, Harlow, UK	GST-CB2C	1:200
Human CB2	Goat	Santa Cruz	C-15, sc-10073	1.4000
Human CB2	Rabbit	Santa Cruz	H-60, sc-25494	1:500

### Co-localisation studies

In order to further characterise cells with P2X_7 _or COX-2 immunoreactivity, serial sections were double labelled by incubation with a mixture of primary antibodies of either COX-2 and Ferritin or P2X_7 _and Ferritin. Immunoreactions for COX-2 and P2X_7 _were revealed using alkaline phosphatase (APAAP – DAKO Ltd) to give a red product, whilst Ferritin immunoreactivity was revealed using the nickel-enhanced ABC peroxidase method to give a grey/black product. Double-labelled preparations were air dried without counterstaining and mounted in Vectamount medium (Vector Labs UK).

### Image analysis

To quantify immunoreactivity of the various markers, computerized image analysis was performed (SeeScan Cambridge, UK). Images were captured via video link to an Olympus BX50 microscope at × 40 objective magnification so that tissue fully occupied each field, and scanned by the computer. Positive immunostaining was highlighted by setting the grey-level detection limits to threshold and the area of highlighted immunoreactivity obtained as % area of the field scanned. Five fields per tissue section were scanned and the mean value used in subsequent statistical analysis.

### Western blot studies

Spinal cord from 6 control and 7 MS patients was available for Western blotting. Spinal cord extracts and mouse macrophage control extracts (BD Biosciences, Oxford, UK) were processed for Western blotting as described [31]. To ensure that similar amounts of protein were loaded onto gels, protein extracts (20 μg; measured by the Bradford dye-binding protein assay, Bio-Rad laboratories, Hertfordshire, UK) were loaded and protein bands visualised by staining membranes with 0.1% (w/v) Ponceau S in 1% (v/v) acetic acid. Only lanes that had similar protein profiles were used for subsequent Western blot analysis. Positive control for the Western blots was mouse macrophage (BD Biosciences, Oxford, UK) extract which also reacted to give a strong 70 kDa band. The COX-2 antibody was used at a titre of 1:2,000. Optical density readings of autoradiographs were taken using a Digit-X densitometer (X-Ograph Ltd, Wiltshire, UK) evenly illuminated on a photography viewer. Background readings were determined by measuring optical density outside the sample lanes. After subtraction of background, the mean of three consecutive readings of protein immunoreactivity at the 70-kDa positions for each sample lane was obtained. Comparisons of control with MS spinal cord specimens were performed on separate occasions using the same original extracts. Because of inter-gel variation, only gels that were performed on the same day were used in comparison studies. Inter-gel variation was corrected by comparing the optical density of the positive control in each blot and adjusting the optical density readings accordingly. This was less than 10%. Because of loading limitations due to the number of wells on the comb it was necessary to perform Western blots on more than one occasion in order to increase number of patient extracts for statistical validity. Our results and controls were reproducible on each occasion. Data was analysed using Mann Whitney U test using GraphPad Prism software, *P *< 0.05 indicated significance.

### PK11195 binding studies

Autoradiography [32] was performed on unfixed cryostat sections of snap-frozen CNS tissue from human post-mortem material using custom synthesised single enantiomer [^3^H] R-PK11195 (Amersham, UK) which in previous studies has been shown to possess higher affinity for the peripheral benzodiazepine binding sites than the commonly used racemate of PK11195 [33].

## Results

### Control spinal cord

**Figure 1:**
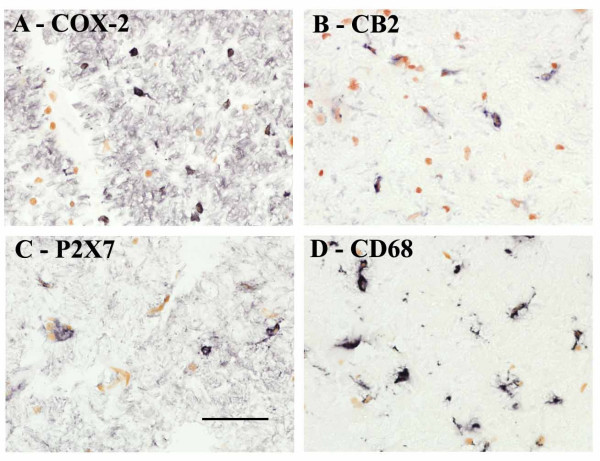
Microglial cells/macrophages in control spinal cord immunostained with antibodies to A) COX-2, B) CB2, C) P2X_7_, and D) CD68. Scale bar = 50 μm.

Antibodies to COX-2, CB2 and P2X_7 _were immunoreactive with scattered, small, nucleated cells some showing fine short processes typical of microglial cells/macrophages (Fig 1A – C). Similar staining was obtained with antibodies to CD68 (Fig. 1D) which is known to be a marker of microglial cells/macrophages.

### Multiple Sclerosis

#### Autoradiography

**Figure 2:**
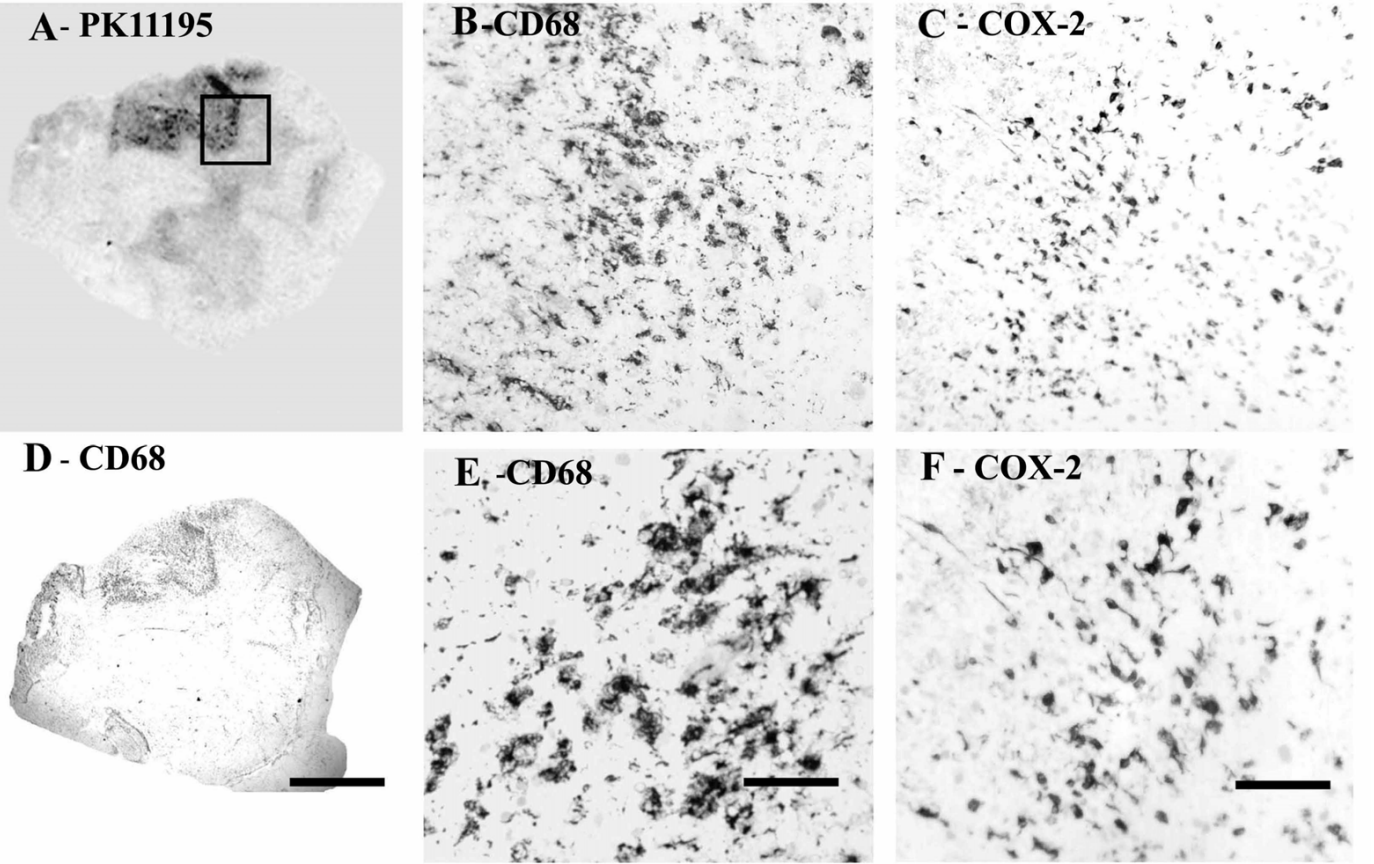
COX-2 immunoreactivity in MS spinal cord is in microglial cells/macrophages. (A) Autoradiographic localisation of ^3 ^[H] PK11195 in a spinal cord from a patient with MS, co-located with CD68 (D) (Scale bars = 1000 μm). The square indicates the area where subsequent CD68 and COX-2 images were taken from. B) And E) Microglial cell/macrophage-like immunostaining with CD68 antibody (Scale bars = 200 μm and 100 μm respectively). C) And F) Microglial cell/macrophage-like immunostaining with COX-2 (Scale bars = 200 μm and 100 μm respectively).

Regional increases of PK11195 binding were found in MS plaques and associated white matter tracts (Fig 2A), and closely matched the spatial distribution of activated microglial cells/macrophages immunostained by CD68 antibodies.

### Immunocytochemistry

#### CD68

Strong microglial cell/macrophage-like staining was seen in affected regions with the antibody to CD68; cells were bipolar with long processes (Fig 2B, 2D and 2E) in both sets of spinal cord specimens. There was no overall difference between the groups: Control spinal cord, n = 7, % area, median and range, 3.54 (2.79–4.99), MS spinal cord, n = 10, 3.92 (2.51–6.68), *P *= 0.6.

#### COX-2

**Figure 3:**
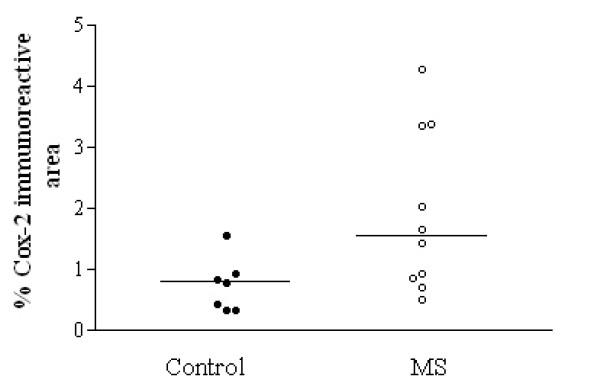
COX-2 immunoreactivity is increased significantly in MS spinal cord. A horizontal line indicates the median value from each group. * P = 0.025

All samples showed glial-like cells, mostly bipolar with short processes scattered throughout the tissue. The processes appeared longer in MS spinal cord, which also appeared to have a greater density of glial-like cells, particularly around plaques (Fig 2C and 2F) and was confirmed by image analysis (Fig 3).

#### CB2

**Figure 4:**
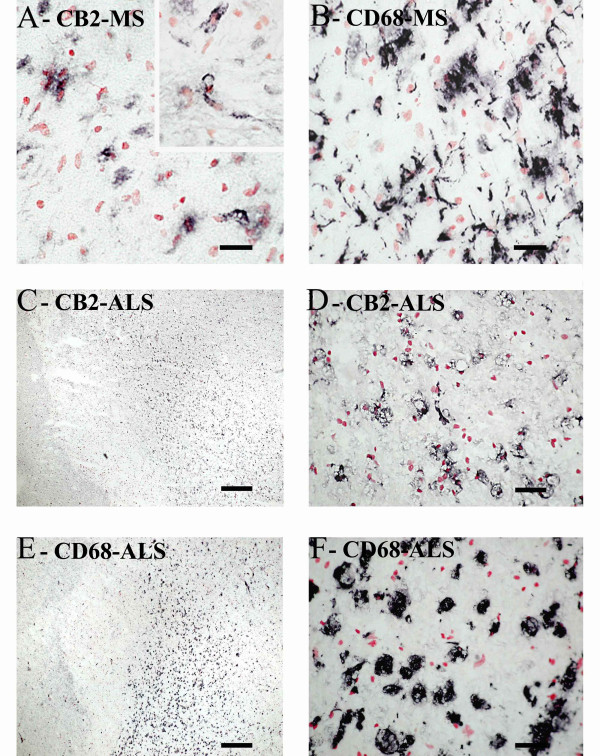
CB2 staining in MS and ALS spinal cord is in microglial cells/macrophages. A) CB2 staining in MS spinal cord. Inset: CB2 staining in parenchymal microglial cells/macrophages. (Scale bar = 50 μm). B) Microglia-like immunostaining with CD68 in the same area as in A (Scale bar = 50 μm). C and D) CB2 staining in ALS spinal cord (Scale bar = 500 μm and 50 μm, respectively). E and F) Microglia-like immunostaining with CD68 (Scale bar = 500 μm and 50 μm, respectively).

**Figure 5:**
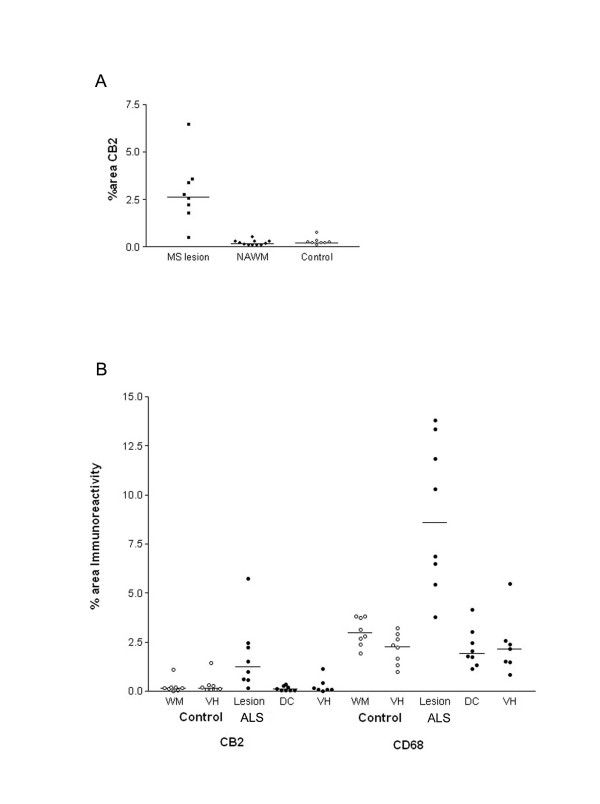
CB2-immunoreactivity is increased in MS and ALS spinal cords. A) Mean % area of CB2 in spinal cord taken from MS (with and without lesion, filled squares and circles respectively) and control spinal cord (open circles) using computerized image analysis. A horizontal line indicates the median value from each group. B) Mean % area of CB2 in ALS spinal cord and CD68 immunoreactivity taken from control and ALS spinal cord using computerized image analysis. A horizontal line indicates the median value from each group, WM, white matter; VH, ventral horn; DC, dorsal column; NAWM, non affected white matter.

Weak CB2 -immunoreactivity was detected in control spinal cord as, small, glial/ macrophage-like cells with no processes, which were scattered throughout the tissue, whilst MS specimens with lesions had a greater density of such glial-like cells, observed with both CB2 antibodies. These were more frequent in the white matter in MS sections with lesions, appeared in clusters, usually in or on the edge of areas of plaques (Fig 4A). CB2 staining in MS spinal cord specimens with no apparent lesions appeared similar to control cord. CB2 staining (Santa Cruz antibody, C-15, sc-10073) in specimens with lesions was increased significantly as measured by image analysis (*P *= 0.0003; Fig 5A). This immunoreactivity was completely abolished when antibody was pre-absorbed with corresponding peptide (available for Santa Cruz antibody only).

Strong microglial cell/macrophage-like staining was seen with the antibody to CD68; cells were bipolar with long processes in both sets of spinal cord specimens (Fig 4B). There was no difference between the control and MS specimens with no apparent lesions: control spinal cord, n = 8, % area, median and range, 2.979 (1.934–3.824), MS spinal cord no lesion, n = 18, 4.155 (2.46–11.74), *P *= 0.0628. However, there was a difference in the MS cord with lesions 12.67 (8.88–15.75), P = 0.012 compared to controls.

### Western blotting

**Figure 6:**
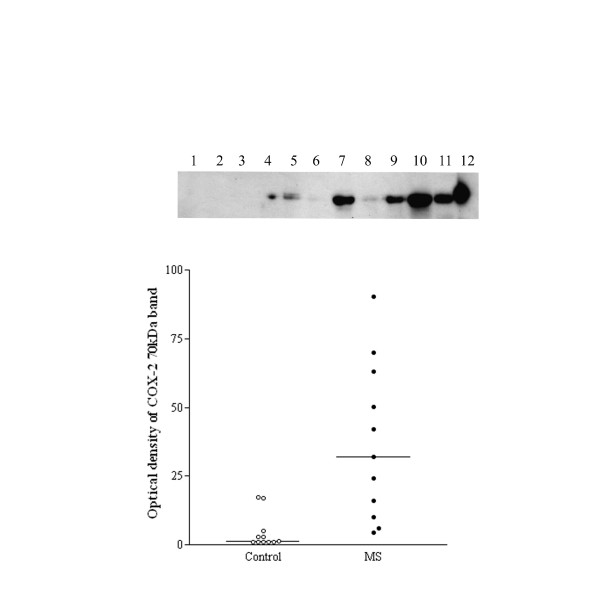
Top panel, representative Western blots of the COX-2 70 kDa band in control (lanes 1–6) and MS (lanes 7 – 11), and lane 12 mouse macrophage positive control. Bottom panel, optical density analysis of COX-2 Western blots. Relative optical density readings of COX-2, 70-kDa bands in the spinal cord of controls compared to MS specimens.

**Figure 7:**
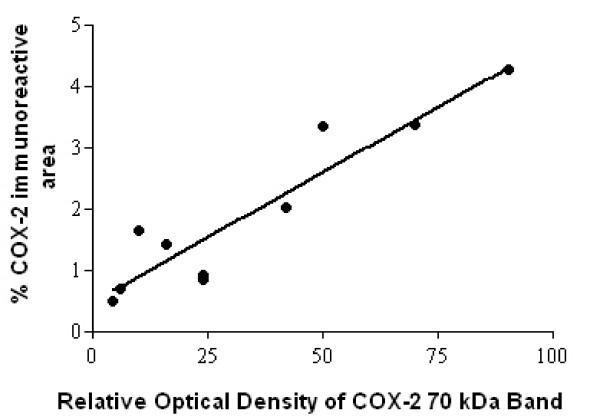
COX-2 Western blots correlate with immunohistochemistry studies. Spearman plots of COX-2, 70 kDa optical density readings compared to COX-2 image analysis from MS spinal cords. Number of XY pairs 10.

A 70 kDa COX-2 band was observed in mouse macrophage control and human spinal cord extracts, which was clearly more prominent in the MS spinal cords (Fig 6). There was good correlation between the COX-2 % area and the optical density of the COX-2 70-kDa band obtained previously in the MS group, Spearman r = 0.89, P = 0.0011 (Fig 7). There was no significant correlation between the COX-2 readings in the control spinal cord group, n = 7, Spearman r = -0.3152, P = 0.5.

### P2X7

**Figure 8:**
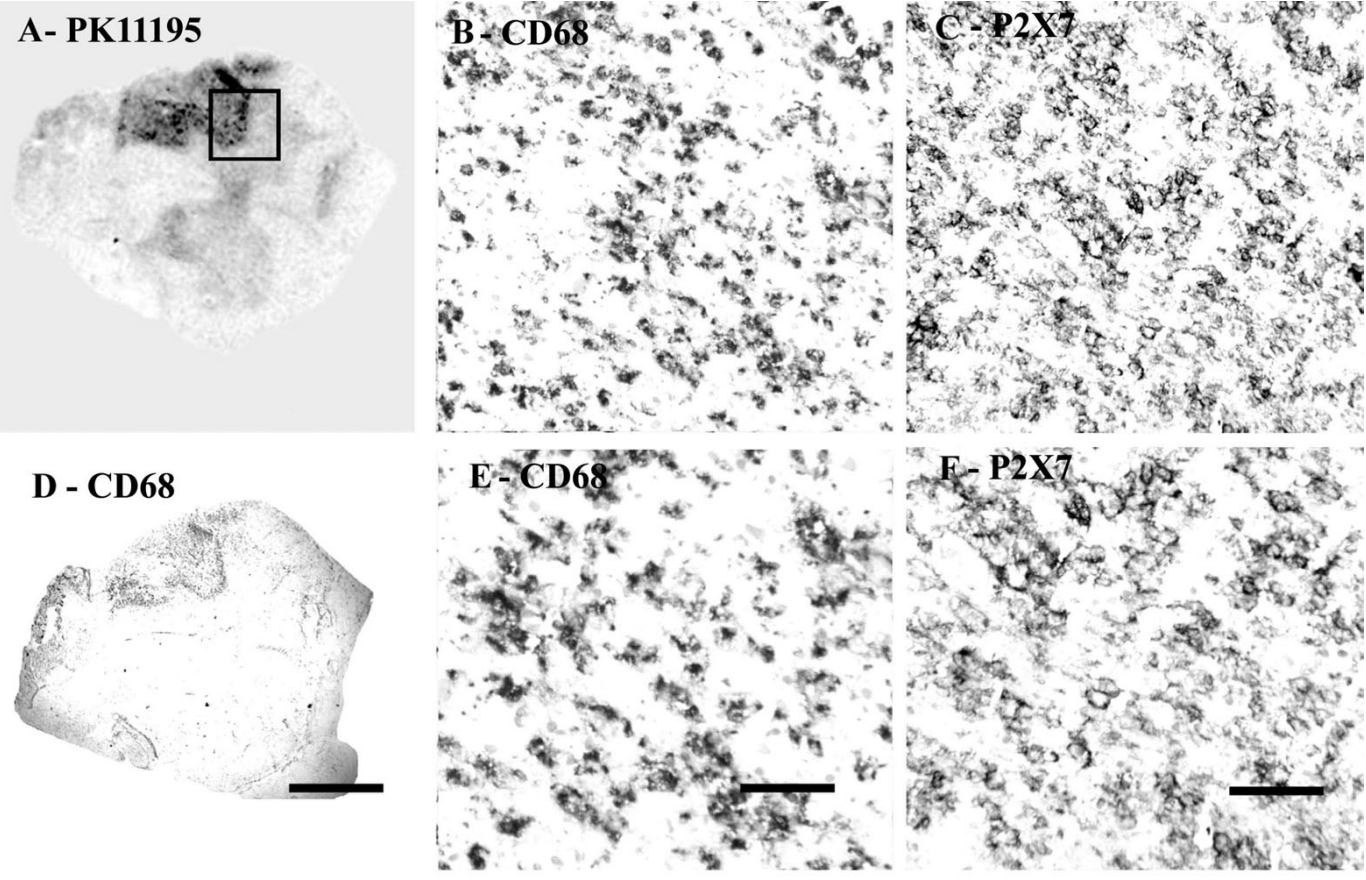
P2X_7 _immunoreactivity is found in microglial cells/macrophages of MS spinal cord. (A) Autoradiographic localisation of ^3 ^[H] PK11195 in a spinal cord from a patient with MS co-located with CD68 (D) (Scale bars = 1000 μm). The square indicates the area where subsequent CD68 and P2X_7 _images were taken from. B) And E) Microglial cells/macrophage -like immunostaining with CD68 antibody (Scale bars = 200 μm and 100 μm respectively). C) And F) Microglial cells/macrophage -like immunostaining with P2X_7 _(Scale bars = 200 μm and 100 μm respectively).

**Figure 9:**
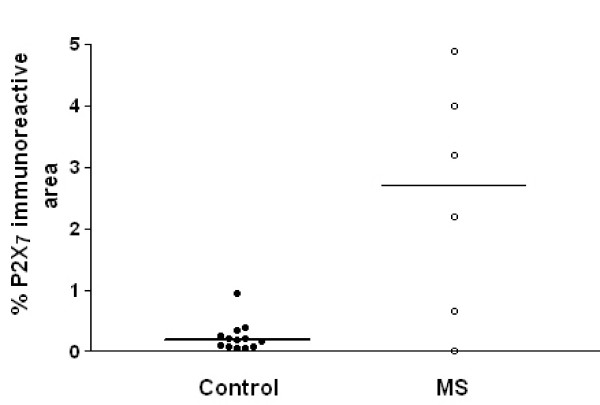
P2X_7 _immunoreactivity is increased in MS spinal cord. Mean % area of P2X_7 _immunoreactivity taken from control and MS spinal cord using computerized image analysis. A horizontal line indicates the median value for each group.

P2X_7 _immunoreactivity was detected mostly in white matter in cells having the appearance of microglial cells/macrophages (Fig 8C and 8F). Immunoreactivity in macrophages was strongest at the cell surface membrane. Immunostaining of serial sections with antibodies to the macrophage marker CD68 (Fig 8B, 8D, 8E) showed very similar staining patterns. MS cases showed P2X_7_-immunoreactive macrophages in clumps/plaques with an accumulation of cells in and around blood vessels. There was a significant (P = 0.032) increase of P2X_7 _-immunostaining in MS cords (Fig 9).

### Co-localisation studies

**Figure 10:**
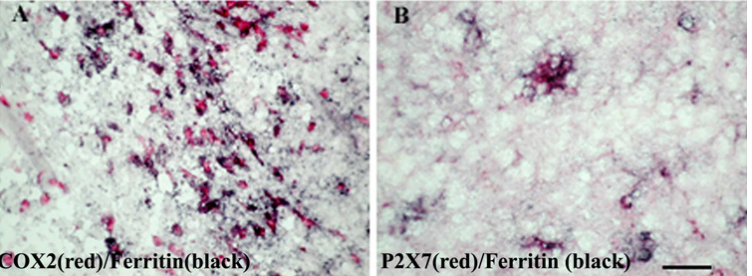
Co-localisation studies of COX-2 and P2X_7 _in MS spinal cord. Double staining of COX-2 or P2X_7 _cells with the microglia marker ferritin in MS spinal cord. Sections were incubated with mixture of A) Ferritin (black) and COX-2 (red) antibodies or B) ferritin (black) and P2X_7 _(red). The majority of COX-2 or P2X_7 _immunoreactive cells appear to be microglial cells/macrophages. Scale bar = 100 μm.

Immunostaining with a mixture of antibodies to ferritin and COX-2 or P2X_7 _showed that the majority of cells with red (P2X_7 _– Fig 10A; COX-2 – Fig 10B) immunoproduct also contained black immunoproduct (ferritin), indicating the presence of P2X_7 _and/or COX-2 in microglial cells/macrophages.

### Amyotrophic lateral sclerosis

#### Autoradiography

**Figure 11:**
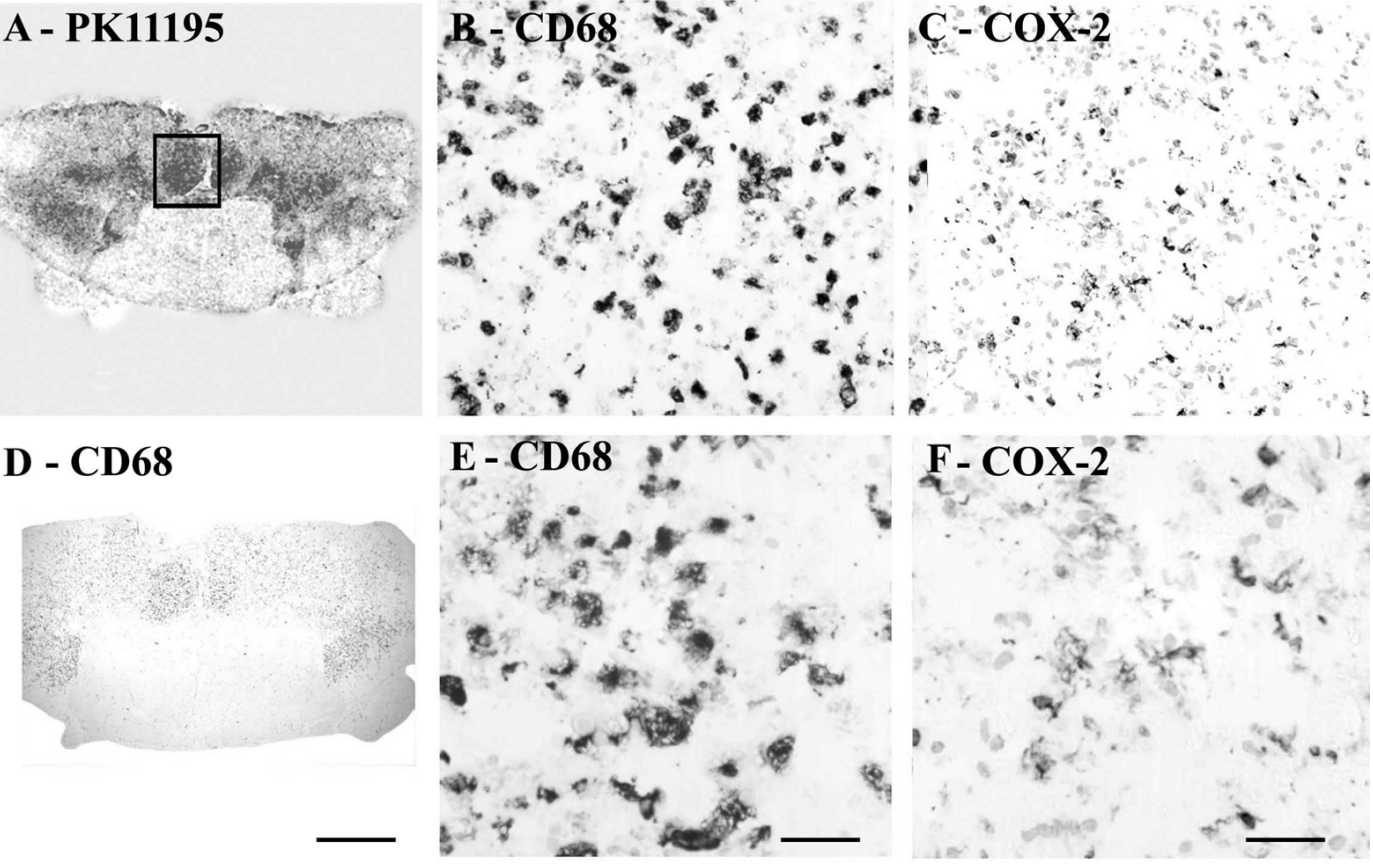
COX-2 immunoreactivity in ALS spinal cord found in microglial cells/macrophages predominantly in white matter. (A) Autoradiographic localisation of ^3 ^[H] PK11195 in a spinal cord from a patient with ALS co-located with CD68 (D) (Scale bars = 1000 μm). The square indicates the area where subsequent CD68 and COX-2 images were taken from. B) And E) Microglial cells/macrophage -like immunostaining with CD68 antibody (Scale bars = 200 μm and 100 μm respectively). C) And F) Microglial cells/macrophage-like immunostaining with COX-2 (Scale bars = 200 μm and 100 μm respectively).

Regional increases of ^3 ^[H] PK11195 binding were found along the corticospinal tract region and closely matched the distribution of activated microglial cells/macrophages immunostained by CD68 antibodies (Fig 11A, B, D, E).

### Immunocytochemistry

#### COX-2

**Figure 12:**
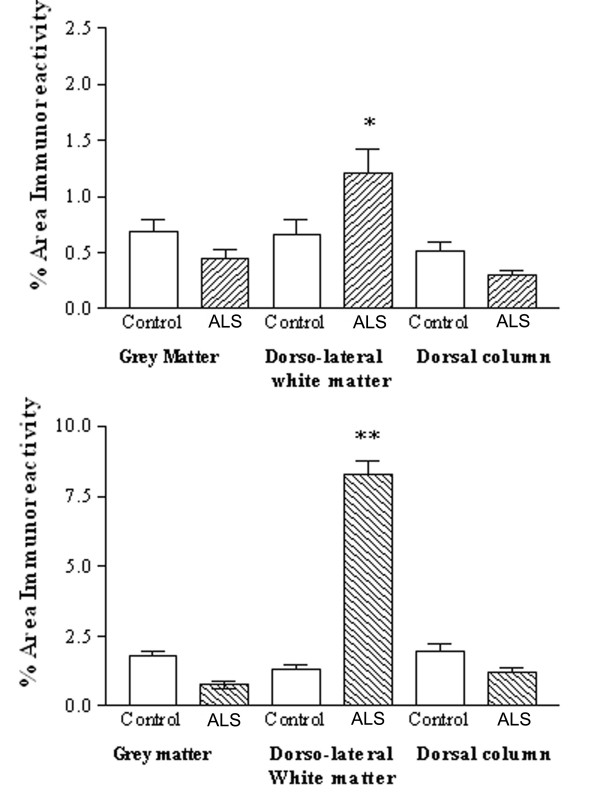
COX-2 is increased in the dorsolateral white matter of ALS spinal cord. Top, Mean % area of COX-2 immunoreactivity in spinal cord (open box, control, shaded ALS) and bottom, Mean % area of CD68 immunoreactivity in spinal cord. Mean ± SEM * *P *= 0.0012, ** *P *= 0.0047.

All samples showed glial-like cells, mostly bipolar with short processes scattered throughout the tissue. The process appeared slightly longer in ALS spinal cord (but not as long as MS cord) and also appeared to have greater density of glial-like cells compared to controls (Fig 11C, 11F). This was confirmed in dorsolateral white matter but not in the grey matter or dorsal columns by image analysis (Fig 12, top). In contrast there was no difference between these regions in control cord.

#### CB2

In all amyotrophic lateral sclerosis cord very intense CB2 microglial cell/macrophage-like staining, associated with long processes, was localized in the dorsolateral white matter, in the region of the degenerating corticospinal tracts (Fig 4C and 4D). Image analysis of these lesions in ALS cords, using the GSK antibody, showed a significant increase of CB2 compared to the dorsolateral white matter of controls (P = 0.0104), and within both the dorsal columns (P = 0.0011) and grey matter (P = 0.0059) of ALS cords (Fig 5B). In contrast there was no difference between these regions in control cord. Similar staining of CB2 in activated microglial cells/macrophages in ALS cord was also observed with the Santa Cruz antibody H-60, sc-25494 (data not shown).

#### CD68

In all ALS cord very intense microglial cell/macrophage-like staining associated with long processes were seen with the antibody to CD68 at a titre of 1:750. Staining was almost exclusively localized to the dorsolateral white matter (Fig 8D and 11D) and significantly increased compared to grey matter in ALS (P = 0.0022) and dorsolateral white matter of controls (P = 0.0012) using image analysis (Figure 12, bottom).

#### P2X7

**Figure 13:**
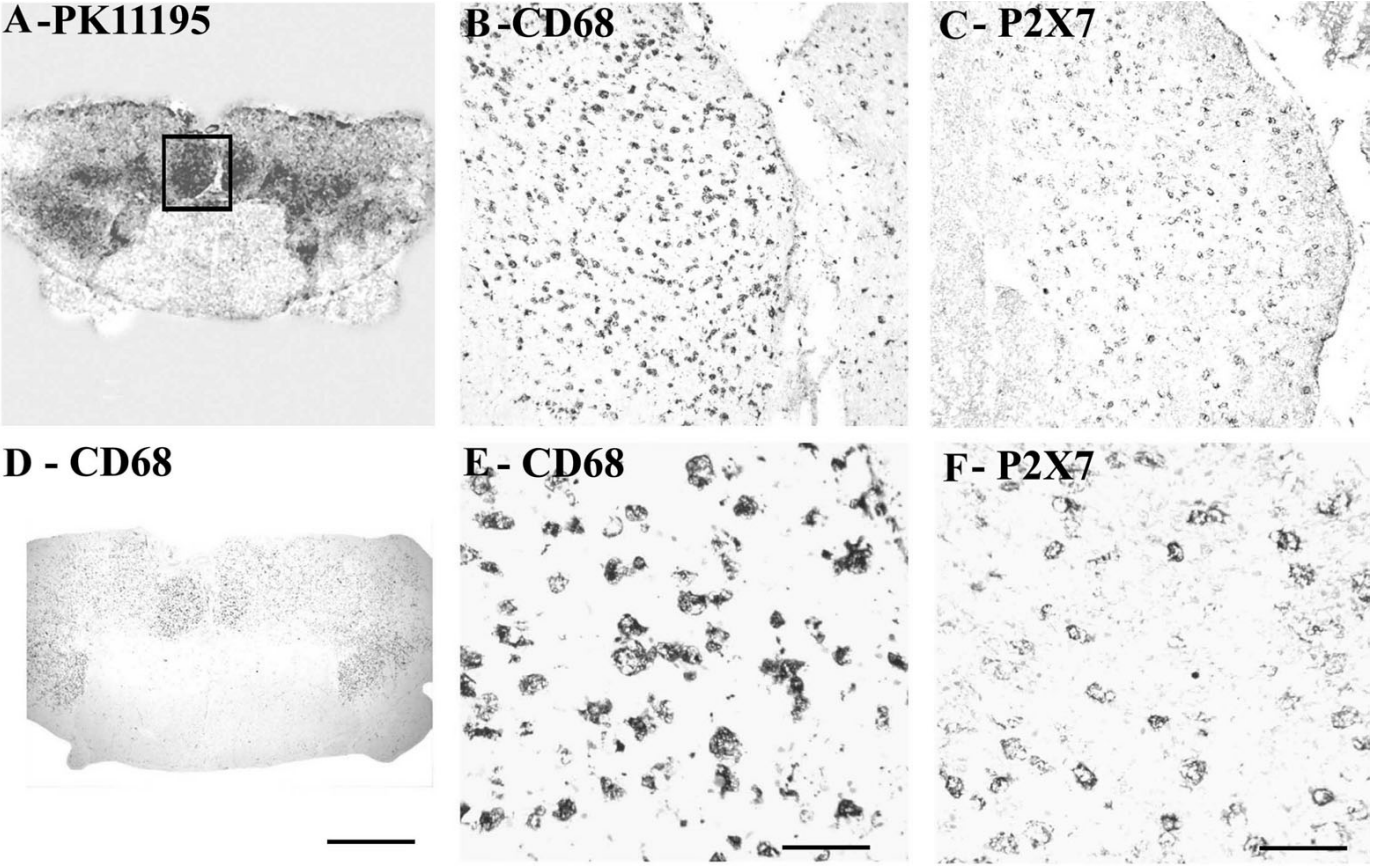
P2X_7 _immunoreactivity localised to microglial cell/macrophage-like cells in ALS spinal cord. (A) Autoradiographic localisation of ^3 ^[H] PK11195 in a spinal cord from a patient with ALS co-located with CD68 (D) (Scale bars = 1000 μm). The square indicates the area where subsequent CD68 and P2X_7 _images were taken from. B) And E) Microglial-like immunostaining with CD68 antibody (Scale bars = 200 μm and 100 μm respectively). C) And F) microglial cell/macrophage-like immunostaining with P2X_7 _(Scale bars = 200 μm and 100 μm respectively).

**Figure 14:**
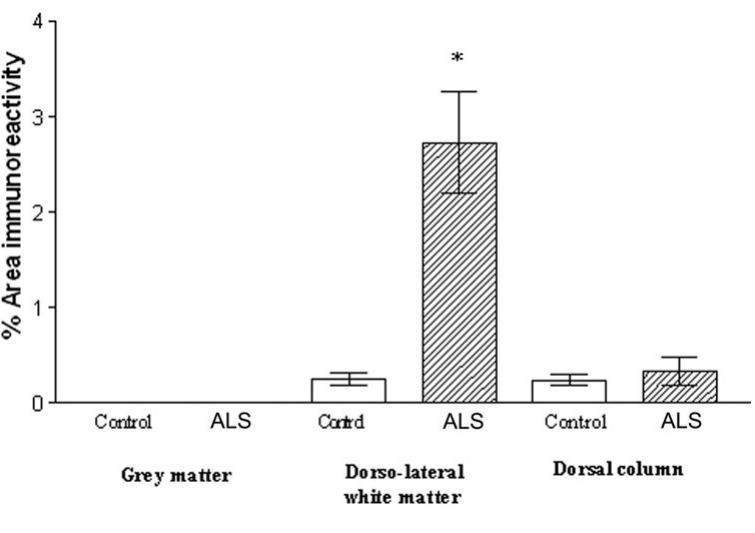
P2X_7 _is increased in the dorsolateral white matter of ALS spinal cord. Mean % area of P2X_7 _immunoreactivity in spinal cord (open box, control, shaded ALS) * P = 0.0001.

**Figure 15:**
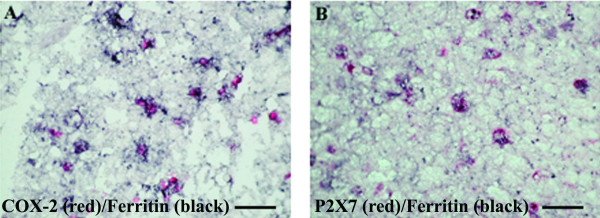
Co-localisation studies of COX-2 and P2X_7 _in ALS spinal cord. Double staining of COX-2 or P2X_7 _cells with ferritin (microglia marker) in ALS spinal cord. Section incubated with mixture of A) ferritin (black) and COX-2 (red) antibodies or B) ferritin (black) and P2X_7 _(red). All COX-2 or P2X_7 _immunoreactive cells are microglial cells/macrophages, scale bars = 100 μm.

All ALS cases showed large numbers of P2X_7_-immunoreactive microglial cell/macrophage-like cells particularly in the dorsolateral white matter and in some cases in anterior columns (Fig 13C, 13F – P2X_7_; Fig 13B, 13E – CD68). These cells were significantly increased in the white matter of ALS cord P = 0.0001 (Fig 14). In most cases of control spinal cord only occasional, P2X_7_-immunoreactive macrophages were detected in contrast to diseased spinal cord that showed cells in abundance. There was relatively negligible P2X_7_-immunoreactivity in the grey matter from either control or ALS cord. Double labelling with antibodies to the microglial cell/macrophage marker, Ferritin showed P2X_7 _and COX-2 present in microglial cells/macrophages (Fig 15A – COX-2/ferritin; 15B – P2X_7_/ferritin).

## Discussion

The studies reported here demonstrate that MS spinal cord contained increased levels of COX-2, CB2 and P2X_7 _receptor in affected regions compared to control spinal cords. The immunohistochemical studies showed a greater number of microglial cells/macrophages containing COX-2 and CB2 immunoreactivity in MS compared to control spinal cord. In MS cord, both COX-2 and CB2 -immunoreactive microglial cells/macrophages often had long processes, particularly when located within active plaques.

In ALS spinal cord, COX-2 was also elevated in regions known to be affected in this condition when compared to control spinal cord, as previously reported [11]. However, in our study, we find the increased COX-2 in microglial cells/macrophages, and not in grey matter or neurons. The increased COX-2-immunoreactive microglial cells/macrophages were localized in the affected regions of the spinal cord, such as dorsolateral white matter with corticospinal tract degeneration, and not in the dorsal columns, which are spared in this condition. In other studies Cox-2 mRNA and protein were found to be significantly elevated in ALS cord, but the location of this increase was not reported [10]. COX-2 over-expression in the spinal cord of patients with ALS has also been shown to be present in neurons and glial cells of the CNS [34] and some reports have shown that COX-2 immunoreactivity in normal rats is localised to neurons of laminae II-III, motoneurons of lamina IX and glial cells [6,35]. In support of this we have found COX-2 immunoreactivity in our studies of human and rat peripheral nerves using the same methods and antibodies as the present study [36]. We did not detect neuronal associated COX-2 immunoreactivity in any of the spinal cord specimens studied including the control spinal cords, in which only a few microglial cells/macrophages with few processes were COX-2 -immunoreactive. These contrasting results between laboratories may reflect differences in methods and/or reagents. In rats, induction of COX-2 mRNA expression in the spinal cord has been demonstrated after intraspinal injections of IL-1α [37] or after mechanical injury to the spinal cord [38]. COX-2 immunoreactivity was also found in vascular endothelial cells and glial cells after IL-1α challenges [37].

Glial activation, including microglial cells/macrophages, is a major histopathological feature of MS and ALS, and may reflect the severity of the disease process. The precise mechanisms of glial activation in these diseases are not fully understood; recent observations implicate a purinergic-signaling pathway [39]. Our findings show that P2X_7 _is up regulated in activated microglial cells/macrophages. A cascade may be postulated – cell death raises extracellular ATP [40], which activates P2X_7 _expressed by microglial cells/macrophages; the latter release IL1β, which in turn induces COX-2. IL1β and PGE2 lead to further cell death and ATP release, and the cycle is perpetuated. In addition, recent reports suggest that ATP, as well as activating quiescent microglial cells/macrophages [41], is able to act as a chemoattractant for microglial cells/macrophages [42], directing them to the source of injury. This, coupled with the release of superoxide from microglial cells/macrophages following P2X_7 _ligation [43] suggests a pivotal role of microglial cells/macrophages in the degenerative processes associated with these diseases. While constitutive expression of P2X_7 _and COX-2 have been reported in different cell types in the brain [6], our data suggest that increased expression of these molecules in MS and ALS occurs predominantly within activated microglial cells/macrophages. In accord with the acknowledged role of microglial cells/macrophages as the brain's endogenous immune effector cells, their presence in inflammatory and degenerative processes may underlie the commonality of pathological mechanisms, which may be targeted to modify disease progression, irrespective of the primary cause.

It has been shown recently that peripheral benzodiazepine receptor ligand PK11195 inhibits the lipopolysaccharide-induced COX-2 expression in human microglial cells/macrophages [44]. This suggests that the binding of this ligand to the PBR site on the mitochondrial outer membrane of microglial cells/macrophages could serve an anti-inflammatory function in the CNS. Thus, the development of drugs that can bind the mitochondrial outer membrane of microglial cells/macrophages, or even the peripheral benzodiazepine receptor ligand PK11195 itself, may be of use in the treatment of some patients with MS and/or ALS. Inhibition of COX-2 has indeed been shown to prevent or attenuate disease progression, in an ALS animal model [12].

The finding of CB2 in microglial cells/macrophages in human spinal cord is of interest as cannabinoids have been shown in animal models to regulate microglial cell/macrophage cell migration [45]. These authors demonstrated a cannabinoid signalling system involving recruiting microglial cells/macrophages towards dying neurons. Of particular interest to the present study, they demonstrated that activated microglial cells/macrophages expressed CB2 receptors at the leading edge of lamellipodia, consistent with the involvement of microglial cells/macrophages in cell migration. Various synthetic cannabinoids have been shown to suppress experimental autoimmune encephalomyelitis (EAE, an animal model of MS) by suppressing inflammation in brain and spinal cord of treated animals [46], including amelioration of both tremor and spasticity in diseased mice [47]. Suppression of EAE by cannabinoids may be related to their effect on corticosterone secretion [48].

Chronic pain models (CCI or Bennett and Chung models, sciatic nerve and spinal nerve ligation, respectively) associated with peripheral nerve injury but not inflammatory chronic pain models (Freund's complete adjuvant injection in the paw) have been shown to induce CB2 mRNA expression in rat lumbar spinal cord on activated microglial cells/macrophages, in regions undergoing neuronal damage [49]. Treatment of rats infected with Theiler's virus (a murine model of multiple sclerosis), with the synthetic cannabinoids WIN 55,212-2, ACEA and JWH-015 during established disease significantly improved the neurological deficits of this disease and reduced microglial cell/macrophage activation in the spinal cord [50,51]. Recently, the selective CB2 receptor agonist AM1241 was shown to reverse tactile and thermal hypersensitivity produced by ligation of the L5 and L6 spinal nerves in rats [52]. This agonist also blocked spinal nerve ligation- induced tactile and thermal hypersensitivity in CB1 knock-out mice, suggesting a mechanism leading to the inhibition of pain that targets receptors outside the CNS. Recently, in CB1 knock out-mice with acute EAE had more prolonged calcium fluxes induced by NMDA agonists and kanic acid induced seizures and death occurred at much lower doses in these animals [53] compared to controls. This suggests that cannabinoids may reduce the excitotoxic effects of acute inflammation on axons. Cannabinoids are predicted not to alter relapse frequency but to reduce the disability acquired as a result of each relapse often seen in MS.

In rats, local administration of the selective CB2 agonist JWH-133 induced a significant regression of malignant tumours generated by inoculation of C6 glioma cells. Evidence of the involvement of CB2 demonstrated, included, the prevention by CB2 but not CB1 antagonists and down-regulation of the CB2 receptor but not CB1 in the tumours themselves. Selective CB2 agonists also induced a considerable growth inhibition on both malignant tumours and tumour vascularization, generated by inoculation of epidermal tumour cells into mice.

CB2 cannabinoid specific agonists have a spectrum of effects that make them very promising candidates for the treatment of pain and, in view of our findings, CNS inflammation and neurodegeneration. In preclinical studies, they inhibit signs of acute nociceptive, inflammatory and neuropathic pain. Because of their peripheral distribution they are predicted not to cause the CNS effects associated with CB1 agonists in normal subjects [54]. Furthermore, it may be possible to up-regulate endogenously produced cannabinoids such as anandamide and 2-AG to mediate therapeutic benefit.

## Conclusion

We conclude that selective CNS-penetrant COX-2 and P2X_7 _inhibitors and CB2 agonist deserve evaluation in the progression of MS and ALS.

## Abbreviations

COX-2 = cyclooxygenase-2; MS = multiple sclerosis; ALS = amyotrophic lateral sclerosis

## Competing interests

The author(s) declare that they have no competing interests.

## Authors' contributions

YY participated in the experimental immunohistochemistry, Western blotting work, analysis of data, drafted the manuscript. PF participated in the experimental immunohistochemistry work and analysis of data. PD participated in the experimental immunohistochemistry work. AN, IPC, and CB were responsible for the design and production of the CB2, P2X_7 _and COX-2 antibodies used, helped with interpretation of the data, and with writing the manuscript. IPC was responsible for the design and production of the P2X_7 _antibodies used, helped with interpretation of the data, and writing the manuscript. AN was responsible for the design and production of the COX-2 antibodies used, and helped with interpretation of the data. CB participated in the conception of the study, development of antibodies, and interpreting the data. RRB participated in the analysis and interpretation of the data, and developing the autoradiographic studies. PA conceived the study and participated in its design and coordination, interpretation and completion of the manuscript. All authors read and approved the manuscript.

## Pre-publication history

The pre-publication history for this paper can be accessed here:


